# Tracing cross species transmission of *Mycobacterium bovis* at the wildlife/livestock interface in South Africa

**DOI:** 10.1186/s12866-020-01736-4

**Published:** 2020-03-04

**Authors:** Petronillah R. Sichewo, Tiny M. Hlokwe, Eric M. C. Etter, Anita L. Michel

**Affiliations:** 1 Department of Veterinary Tropical Diseases, Bovine Brucellosis and Tuberculosis Research Programme, Faculty of Veterinary Science, University of Pretoria, Pretoria, Republic of South Africa; 2 Department of Animal Sciences, Faculty of Natural Resources Management and Agriculture, Midlands State University, Gweru, Zimbabwe; 3 Diagnostic Services Programme, ARC-Onderstepoort Veterinary Research, Pretoria, Republic of South Africa; 4 Department of Animal Production Studies, Faculty of Veterinary Science, University of Pretoria, Pretoria, Republic of South Africa; 5 CIRAD, UMR Animal, Santé, Territoires, Risque et Ecosystèmes (ASTRE), Montpellier, France; 6 ASTRE, Univ Montpellier, CIRAD, INRA, Montpellier, France; 7 Research Associate at the National Zoological Gardens of South Africa, Pretoria, South Africa

**Keywords:** African buffalo, Bovine tuberculosis (bTB), Cattle, *Mycobacterium bovis* (*M. bovis*), Strains, Wildlife, Wildlife/livestock interface

## Abstract

**Background:**

Bovine tuberculosis (bTB) affects cattle and wildlife in South Africa with the African buffalo (*Syncerus caffer*) as the principal maintenance host. The presence of a wildlife maintenance host at the wildlife/livestock interface acting as spill-over host makes it much more challenging to control and eradicate bTB in cattle. Spoligotyping and mycobacterial interspersed repetitive unit-variable number of tandem repeat (MIRU-VNTR) genotyping methods were performed to investigate the genetic diversity of *Mycobacterium bovis* (*M. bovis*) isolates from cattle and wildlife, their distribution and transmission at the wildlife/livestock interface in northern Kwa-Zulu Natal (KZN), South Africa.

**Results:**

SB0130 was identified as the dominant spoligotype pattern at this wildlife/livestock interface, while VNTR typing revealed a total of 29 VNTR profiles (strains) in the KZN province signifying high genetic variability. The detection of 5 VNTR profiles shared between cattle and buffalo suggests *M. bovis* transmission between species. MIRU-VNTR confirmed co-infection in one cow with three strains of *M. bovis* that differed at a single locus, with 2 being shared with buffalo, implying pathogen introduction from most probably unrelated wildlife sources.

**Conclusion:**

Our findings highlight inter and intra species transmission of bTB at the wildlife/livestock interface and the need for the implementation of adequate bTB control measures to mitigate the spread of the pathogen responsible for economic losses and a public health threat.

## Background

*Mycobacterium bovis* (*M. bovis*) is host adapted to cattle where it causes bovine tuberculosis (bTB); but is also a multi-host pathogen that affects other domesticated animals, wildlife and humans [1]. The disease has been nearly eradicated in developed countries, but it is widespread in developing countries where it is considered a risk to veterinary and public health and has a negative economic impact and threatens livelihoods [2–4].

The occurrence of wildlife maintenance hosts has complicated the control of the disease especially at the wildlife/livestock/human interface where there is overlapping of territories due to encroachment of human activities into wildlife conservation areas and potential escape of wildlife from these areas [5–8]. Cross-species transmission of bTB has been documented at the wildlife/livestock interface, with wildlife maintenance hosts such as the African buffalo (*Syncerus caffer*) in South Africa and the Kafue lechwe antelopes (*Kobus leche kafuensis*) in Zambia [9–11]. Several wildlife maintenance hosts have also been recognized in some developed countries as significant sources of infection and make it difficult to control as well as eradicate bTB in cattle [12–17] .

In South Africa bovine tuberculosis affects cattle and has been documented in several wildlife species for example lion, buffalo, cheetah,hyena, impala, kudu, nyala and others [18, 19]. Intra and inter species transmission of *M. bovis* occurs through direct contact (inhalation of contaminated aerosol) or through the consumption of contaminated food or water [5, 20]. Bovine TB is prevalent in the African buffalo in the Hluhluwe iMfolozi Park (HiP) and spill-over to a range of other wild animal species such as lion, chacma baboon, bush pig and warthog has been reported [17, 21, 22]. Molecular analysis of *M. bovis* isolates from wildlife has revealed spoligotype patterns that are being shared with communal cattle thereby suggesting cross-species transmission of bovine tuberculosis at the wildlife/livestock/human interface [18]. In addition, the absence of an effective bTB control programme in cattle, consumption of contaminated raw animal products and a high prevalence of HIV/AIDS exacerbates the risk of zoonotic TB in communities living at the wildlife/livestock/human interface [1, 23–25].

Genotyping methods are used in epidemiological studies to trace the origin of infections, understand the circulation of the pathogen in particular populations and provide information of transmission pathways [26]. The data from these studies are useful for the development of functioning bTB control and management programs especially at the wildlife/livestock/human interface [6, 21]. Spoligotyping and MIRU-VNTR are genotyping techniques that have been recently adopted for use in molecular epidemiology for the discrimination of isolates in the *Mycobacterium tuberculosis* complex based on the analysis of the repeated sequences in the bacterial genome [27]. These methods are reliable and rapidly provide information on the genetic structure, identification of epidemiological links and the pattern of disease occurrence [27].

Spoligotyping, uses polymerase chain reaction (PCR) and reverse-hybridization blotting to evaluate the genetic diversity of *M. tuberculosis* complex isolates using the Direct Repeat locus (DRs) and its unique spacer sequence for the differentiation of these organisms [28]. The results reveal the presence or absence of individual spacer sequences and can be compared with an international database of spoligotype (www.Mbovis.org) [29]. The MIRU-VNTR technique, characterizes mycobacterial interspersed repetitive units (MIRU) based on the variable number of tandem repeats [27]. Research groups have identified different combinations of VNTR loci for typing of *M. bovis* and variation according to geographical location has been documented hence, there is a need for a specific panel of loci in a region [30].

The study area is a communal farmland that is surrounded by public and private game reserves where bTB has been previously reported in wildlife, such as the Hluhluwe iMfolozi Park (HiP), Munyawana and Mkhuze game reserves [22, 31]. Recently bTB was detected in traditionally farmed cattle in this wildlife/livestock interface [32]. Currently, there is inadequate information on the inter species transmission of bTB between the game reserves and neighbouring communities. Therefore, the aim of the study was to investigate the population structure of *M. bovis* strains in wildlife and cattle and assess the transmission of bTB across these species at this wildlife/livestock interface.

## Results

### Spoligotyping

**Fig. 1:**
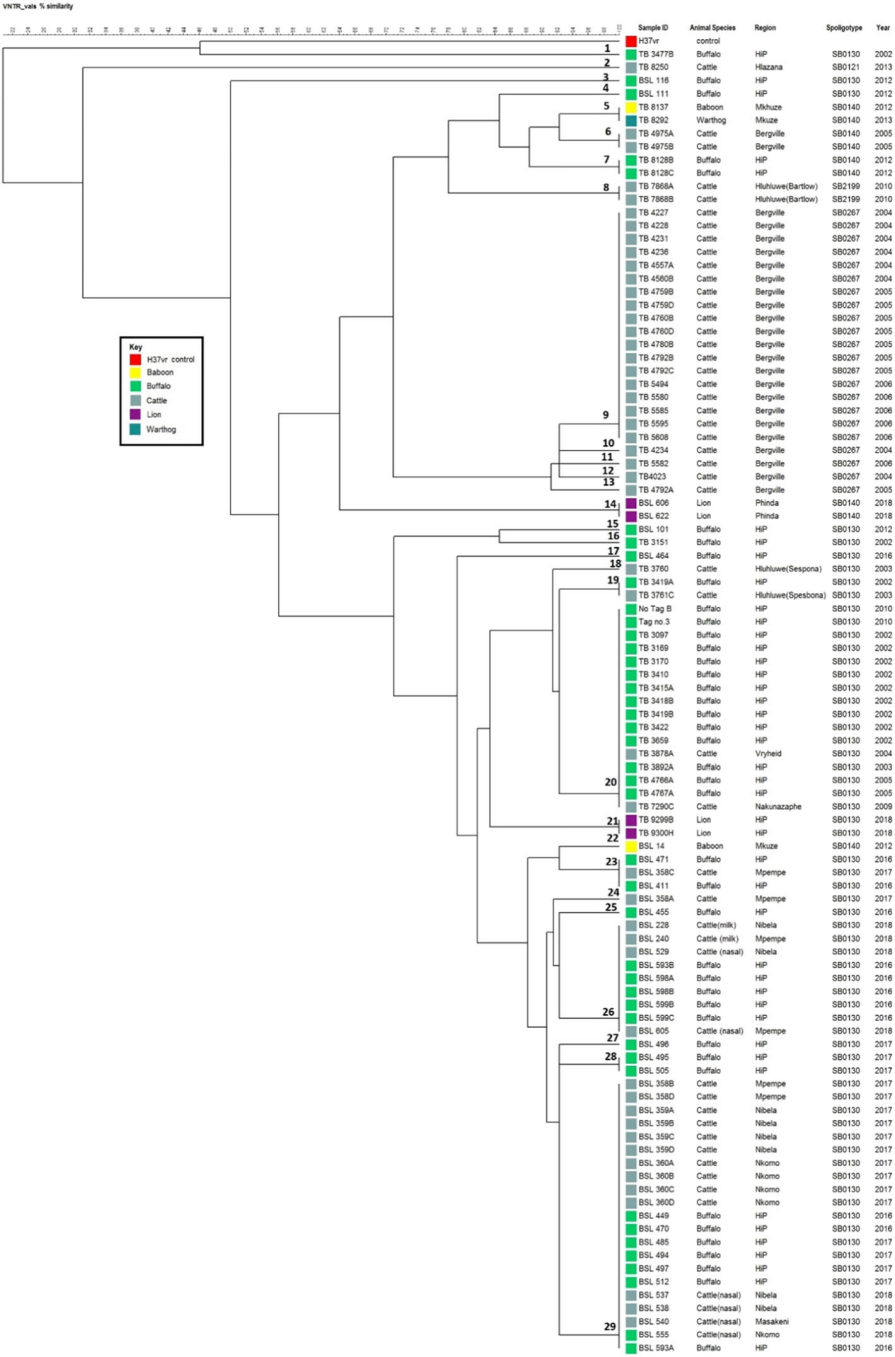
Dendrogram showing genetic relationships of *M. bovis* isolates from cattle registered at dip tanks (Nibela, Nkomo, Masakeni, Mpempe, Hlazana, Nakunazaphe), cattle from various farms in KZN (, Bergville), isolates from bTB infected wildlife from Hluhluwe iMfolozi Park (HiP), Mkhuze and Munyawana game reserves. Colours are for visual differentiation of different animal species. The spoligotype was also included for each isolate. VNTR profiles are labelled from 1 to 29. The isolates corresponded to individual animals identifiable by a unique BSL or TB number

A total of 99 *M. bovis* isolates were obtained from culture of wildlife and cattle samples. Fifty-one (51) isolates were derived from cattle, 41 isolates from buffalo, 4 isolates from lion, 2 isolates from baboons and one from a warthog. The isolates were confirmed as *M. bovis* as they lacked spacer 3,9,16 and 39–43 in the spoligotype patterns which is a feature that is used to distinguish *M. bovis* from *Mycobacterium tuberculosis* (*M. tuberculosis*). A total of five spoligotype patterns were identified. The predominant pattern was SB0130 that lacked additional spacer 11. Out of the five spoligotype patterns, two spoligotypes were shared between cattle and wildlife (SB0130; SB0140); SB2199, SB0121 and SB0267 were found in cattle only as shown in Fig. 1. Spoligotype SB0140 was identified in isolates from baboons and warthog from Mkhuze game reserve, lions from Munyawana game park, African buffaloes from HiP and cattle from a farm near the town of Bergville. The spoligotype patterns were characterised by a lack of additional spacers and named according to the corresponding spoligopatterns found in the international standard database (www.Mbovis.org).

### Mycobacteria interspersed repetitive unit-variable number of tandem repeats (MIRU-VNTR)

Using the 13 loci VNTR panel, 29 distinct genetic profiles were identified from the 5 spoligotypes. The VNTR profiles were designated (genotype)1–29 for identification purposes on the dendogram in Fig. 1. Five VNTR profiles were shared between buffalo and cattle, whilst other wildlife species did not share profiles with cattle. Ten VNTR profiles were exclusively found in buffalo while another set of 10 profiles were detected in cattle only. One VNTR profile was shared between a baboon and warthog, while 2 VNTR profiles and 1 VNTR profile were exclusively found in lions and a baboon, respectively.

SB0130 spoligotype pattern was differentiated into 17 genetic profiles, whereby 5 profiles were shared between cattle and buffalo, 9 being singletons in buffalo, 2 profiles in cattle only and 1 profile in lions only from HiP, as shown in Fig. 1. Co-infection with different strains was detected in tissues from one cow (BSL358) that had 3 genetic profiles that differed at one locus with two of the profiles being shared with different buffaloes (VNTR 23 and 29) and one singleton VNTR 24. Sharing of VNTR profiles was also revealed between cattle from different dip tanks and some farms in the province. VNTR 26 was shared by *M. bovis* isolates from cattle (milk and nasal swabs samples) and tissue samples from buffaloes. VNTR 29 was shared by *M. bovis* isolates from cattle nasal swabs, tissues and buffalo tissue samples.

SB0140 was differentiated into 6 VNTR profiles that included; VNTR 6 in cattle, VNTR 7 in buffalo from HiP, VNTR 5 being shared by a baboon and warthog from Mkhuze game park, one singleton in a baboon (VNTR 22), VNTR 14 and VNTR 21 in lions from Munyawana and HiP game parks, respectively. SB0267 had 5 VNTR profiles (VNTR 9–13) from cattle isolates obtained from a single farm in Bergville as depicted in Fig. 1.

## Discussion

This study investigated the *genetic* diversity of *M. bovis* isolates at the wildlife/livestock interface in northern Kwa-Zulu Natal province, South Africa. The results suggested inter-herd and inter-species transmission based on the spoligotype pattern and VNTR types being shared between cattle from different dip tanks or farms and buffalo.

A high genetic variability of *M. bovis* strains in South African cattle populations has previously been reported [18, 21] and has remained unchanged over more than two decades, as shown in this study. Genetic variability displayed in the VNTR profiles in cattle in our study area (15 VNTR profiles) either infers several local and stable sources of infection or the long-term persistence and evolution of one or more precursor strains. The spoligotypes SB0130 and SB0140 strains are linked to the European 1 (Eu1) clonal complex and where possibly introduced into the country during cattle imports from the UK during colonial times [21, 33]. Thus, the numerous VNTR types might be related to the earlier introduction of bTB into this community; as it has been proposed that persistence of an infection presents more opportunities for genetic evolution [21].

The identical VNTR profiles that were detected in isolates from cattle and African buffalo over the years strongly indicate cross species transmission. High clonality revealed by the isolates indicate the active and recent transmission of *M. bovis* between animal species as previously reported [10]. Hence, the presence of clusters of wildlife and cattle isolates means that related isolates of *M. bovis* are spreading at the wildlife/livestock interface. The presence of *M. bovis* in buffalo is of significance as these represent a maintenance host that can re-infect cattle populations as documented by Musoke et al. (2015) [10]. This is supported by activities that involve wildlife-to-livestock contact at this interface as these have been identified as highly significant risk factors for *M. bovis* infection in cattle [34].

Co-infection was confirmed by the isolation of multiple strains of *M. bovis* in one cow BSL 358 (VNTR 23, 24, 29) with VNTR 23 and 29 profiles being shared with buffaloes. Multiple strain infections demonstrate repeated transmission events caused by several sources of infection or the evolution of ‘old’ strains within the animal populations. The former is in line with the communal production system at the wildlife/livestock interface that is characterised by intermingling of cattle and contact with wildlife at shared resources [34–37]. Hlokwe et al., (2011) reported co-infection of two or more spoligotypes in cattle from the same farm (herd) as an indicator of independent unrelated sources of infections [31]. However, in this study, the VNTR strains detected in the cow (BSL358) shared the same spoligotype and only differed at only one locus suggestive of strain evolution or mutation in the cow following a single infection. A study in Ireland reported co- infection in one badger (three spoligotypes and three VNTR strains that differed at more than one locus) as an indicator of repeated exposure of the animal to various sources of infection hence the numerous *M. bovis* strains [38].

The excretion of *M. bovis* in milk and nasal discharges detected in bTB infected cattle herds facilitates both intra and inter species transmission [25] during direct contact at shared feed or water sources or, in the case of humans, through consumption of contaminated milk. The traditional cattle farming practices that were reported by the farmers in our previous study promote contact of infected herds with uninfected herds at common watering points and pastures [34]. These results conform to the previous findings that reported SB0130 as one of the major spoligotypes that cause *M. bovis* infection in wildlife and cattle in South Africa [18, 21, 31]. In particular, spoligotype SB0130 was dominant in cattle as well as wildlife between 1993 and 2013 [18, 21] and still most frequently isolated in our study (from 2002 to 2018). We believe this trend is an indicator of ineffective control measures that fail to eradicate the strain from cattle, and points to a continuous risk of infection and or re-infection to wildlife and cattle.

The other factors that contribute to the maintenance of the infection are the results of unsuitable animal management practices, for example, free movement of cattle that leads to intermingling of animals with different bTB status, trade of infected animals and inadequate bio-safety measures. As suggested in literature, biological, ecological and anthropological factors influence the transmission of *M. bovis* at the wildlife/livestock interface [9]. At this wildlife/livestock interface introduction of cattle into herds without bTB pre-testing for traditional purposes and the co-grazing of cattle with wildlife as well as sharing of water sources were recognized as risky practices for bTB transmission to cattle [34].

MIRU-VNTR revealed a high genetic variability in the 5 spoligotype patterns using the previously evaluated 13 VNTR locus for South African isolates [39], indicative of its superior discriminatory power over spoligotyping. The slowly mutating marker that is used in spoligotyping indicates the evolutionary history of isolates and is useful in the evaluation of eradication programs. Whilst the more rapidly evolving markers that are used in MIRU-VNTR are suitable for tracking transmission and determining the origins of outbreaks [40]. The use of both spoligotyping and MIRU- VNTR in combination provided a better insight into the epidemiology of bTB and evaluation of the control program, thus suitable solutions could be designed for South Africa.

As reported by Haddad et.al. (2001), a comparative study of *M. tuberculosis* strain diversity between developed and developing countries, concluded that in a high human TB prevalence situation, a dominant spoligotype will most likely exclude other types [41]. Whereas, in a low human TB prevalence situation more *M. tuberculosis* genotypes can be present. This has been established in the context of bTB in cattle with reference to numerous genotypes displayed by *M. bovis* isolates from developed countries where bTB prevalence is low due to successful national control programs [40]. In our study setting where a high bTB was found [32], the SBO130 was dominant in both cattle and wildlife, indicative of a poor efficacy of current control measures in this communal area.

## Conclusion

The findings from this study highlights the significance of wildlife (African buffalo) and cattle in the persistence of *M. bovis* infection at the wildlife/livestock interface. National bTB control programs need not only to focus on cattle but also consider the role of wildlife and their transmission dynamics for complete bTB eradication. Co-operation from all relevant stake holders is necessary for the application of stringent control measures in both traditionally farmed cattle and wildlife to successfully reduce bTB at the wildlife/livestock interface.

## Methods

### Study area

The cattle samples were obtained from animals that were bTB tested during a cross-sectional study that was conducted at 4 dip tanks (Nibela, Nkomo, Mpempe, Masakeni) in Big 5 False Bay Municipality, in uMkhanyakude district, northern KwaZulu-Natal province, South Africa. Bovine TB testing of cattle was previously carried out in September 2016 and March 2017 as part of a One Health investigation into the epidemiology of bTB at the wildlife/livestock/human interface [32]. Additional cattle samples were collected from farms and other dip tanks in the province during bTB outbreaks. Wildlife samples were collected from the surrounding game reserves that include HiP, Munyawana and Mkhuze. The map of the study area has been previously described by (Sichewo et al., (2019) [32].

The study area is defined as a wildlife/livestock/human interface due to the communal farmland being bordered by game farms or reserves where *M. bovis* infection has been diagnosed in African buffalo and other wildlife species [42]. The farmers in this community depend on subsistence agriculture and livestock as their main source of income [43]. Uncontrolled movement of livestock into/near game reserves during the dry seasons or periods of extensive drought is a common practice in the area.

### Sample collection and processing

#### Milk, nasal and tissue samples from cattle

In June and July 2017, 30 milk samples were collected from interferon gamma positive animals that belonged to the 4 dip tanks were bTB testing was previously carried out in September 2016 and March 2017 [32]. An average of 25 ml of milk was collected into 50 ml centrifuge tubes from all lactating animals. A total of 99 nasal swabs were collected from the same bTB infected herds, [32] through random sampling using 50 cm handmade swabs that were placed into phosphate buffer as the transport medium. The milk samples were frozen, and the nasal swabs were placed at 4 °C and transported to the University of Pretoria, Department of Veterinary of Tropical Diseases in a cold chain.

Routine tissue sample submission was carried out by the state veterinary officials between 2002 and 2013 from i) tuberculin skin test and gamma interferon test positive cattle from farms in KZN and ii) suspect lesions detected in cattle during routine slaughter at abattoirs. These were processed following standard operating procedures at the Onderstepoort Veterinary laboratories as outlined by Hlokwe (2014) [18]. In 2017, following slaughter of three test positive cows, one cow from each of the three dip tanks Nibela, Nkomo and Mpempe, appropriate samples were collected. Either the entire lymph node or approximately 5–10 g of tissue were collected from the head, thoracic and mediastinal lymph nodes and, where relevant, from bTB like lesions observed in other organs such as the lungs, liver, spleen, kidney and mammary glands.

#### Wildlife tissue samples

During the annual bTB management culling program in the HiP, tissue samples were collected from skin test or gamma interferon positive buffalo between 2002 and 2017. In addition, samples were collected from other wild animals such as the lion, baboon and warthog in game reserves in KZN province that included HiP, Munyawana and Mkhuze either during i) TB passive surveillance of all wild animals found dead or ii) culling of skin test or gamma interferon positive wildlife. The samples included submandibular, retropharyngeal, tracheobronchial, mediastinal, mesenteric lymph nodes as well as sections of tonsils, lungs and any tissues with granulomatous lesions.

All the tissue samples that were collected from cattle and wildlife were packaged into zip lock bags and transported frozen to the University of Pretoria-Department of Veterinary Tropical Diseases and Onderstepoort Veterinary Research BSL2+ laboratory for mycobacterial culture.

### *Mycobacterium bovis* culture and identification

Raw milk was decontaminated using 1% cetylpyridinium chloride (CPC) and 2% NaCl as previously described by Michel, 2015 [43]. The sediment from the centrifugation was inoculated onto Löwenstein-Jensen (LJ) media with pyruvate and an antibiotic cocktail consisting of polymyxin B (200 IU/ml), amphotericin B (10 μg/ml) carbenicillin (100 μg/ml) and trimethoprim (10 μg/ml) (NHLS, South Africa) and incubated at 37^o^ C for 10 weeks. Decontamination of nasal swabs was done using the modified Petroff method (2% HCl), followed by centrifugation and the sediment treated with amphotericin B (50 μg/ml). The solution was inoculated onto LJ media with pyruvate and the above antibiotics and incubated at 37^o^ C for 10 weeks with weekly monitoring.

The animal tissue samples were processed according to the method previously described by Alexander et al. (2002) [44]. Briefly, tissues samples were decontaminated using two methods with a final concentration of 2% NaOH and 1% HCl. These were inoculated on four slants of LJ media supplemented with pyruvate and incubated at 37^o^ C for up to 10 weeks. The Ziehl-Neelsen (ZN) staining and culture characteristics (growth rate and colony morphology) were used to identify *M. bovis* isolates from tissue, nasal and milk cultures. DNA extraction was performed using the boiling method whereby the isolates were suspended in100 μl of sterile distilled water and heated to 95 °C for 25 min using a heating block. Thereafter the DNA was stored at -20 °C and used in all the subsequent PCR based reactions [39]. *M. bovis* was confirmed using PCR that is performed using primers that target the Region of Difference (RD), RD4 and RD9 for deletion analysis as previously detailed by Warren, 2006 [45]. The distribution of the isolates according to animal species, location and sample type are as shown in Table 1.

**Table 1:** Animal species, their origins and numbers of *M. bovis* isolated

Animal species	Location	Sample type	Number of *M. bovis* isolates
Cattle	Nibela dip tank	organ tissues	4
		nasal swab	3
		Milk	1
	Mpempe dip tank	organ tissues	4
		nasal swab	1
		Milk	1
	Masakeni dip tank	organ tissues	4
		nasal swab	1
	Nkomo dip tank	nasal swab	1
	Nakunazaphe dip tank	organ tissues	1
	Hlazana dip tank	organ tissues	1
	Commercial farm 1	organ tissues	24
	Commercial farm 2	organ tissues	2
	Commercial farm 3	organ tissues	2
	Commercial farm 4	organ tissues	1
Buffalo	HiP game reserve	organ tissues	41
Baboon	Mkhuze game reserve	organ tissues	2
Lion	Munyawana game reserve	organ tissues	2
	HiP game reserve	organ tissues	2
Warthog	Mkhuze game reserve	organ tissues	1
Grand Total			99

### Genotyping of *Mycobacterium bovis* isolates

#### Spoligotyping

Spoligotyping was applied to isolates that were confirmed as *M. bovis* using culture for further differentiation according to the standard protocol described by Kamerbeek 1997 [46]. Briefly, a PCR reaction was carried out to amplify the spacer sequence of the DR locus using primers DRa (biotinylated) and DRb. The spacer sequences were detected by hybridization of the biotin labelled amplified products using ECL against 43 spacer oligonucleotides covalently linked to a membrane. A commercially available kit was used, and the procedure was conducted according to the manufacturer’s instructions (SPOLIGOTB, Mapmygenome, India). A specific pattern of hybridization signals was generated that represented the absence or presence of the spacer sequences presented in a binary code that was translated into a spoligotype octal code according to the established algorithm. The spoligotyping pattern were named following the nomenclature of the international *M. bovis* spoligotype database (www.Mbovis.org).

#### Variable number of tandem repeats

The *M. bovis* isolates were also genotyped by PCR amplification using a panel of 13 VNTR markers previously established for South African isolates by Hlokwe, van Helden and Michel, 2013 [39]. These include 4 ETRs loci (A, B, C, E), 3 MIRUs loci [16, 23, 26], QUBs loci (11a, b,18,26) and 2 M. tubs [12, 21]. The PCR reactions for each marker were carried out separately using specific primer sequences (Inqaba Biotechnical Industries, South Africa). The procedure was carried out as described by Le Flèche, 2002 [47]. In brief, a 20 μl reaction was used for the PCR and it contained 2 μl DNA template, 10 μl Qiagen master mix, 0.5 μl of each of the two 10 μM primers and 7 μl of DNA free water. The PCR cycle of reactions were as follows: initial denaturation at 94 °C for 5 min, followed by 40 cycles of initial denaturation at 94 °C for 1 min, annealing at 62 °C for 1 min and elongation at 72 °C for 1.5 min and the final elongation at 72 °C for 10 min.

The PCR was carried out using the PCR thermocycler machine (2700, Applied Biosystems). The PCR products were separated electrophoretically using 3% agarose gel at 85 V for 3 h. The band sizes were estimated against a 100 bp DNA ladder and converted into copy number that was saved as the digital VNTR profile in Excel [47]. The data was exported into Bionumerics software package version 7 (Applied Maths, Sint-Martens-Latem) as a character data for analysis.

The unweighted pair-group method using average linkages algorithm (UPGMA) was used to construct the relevant dendogram and the categorical coefficient was used to calculate the similarity of the Multi locus VNTR analysis (MLVA) profiles. In this study, a cluster was defined as a group of isolates with 100% genetic similarity. Additional information on the year of sample collection, location of sample collection (dip tank/game reserve/farm), animal species and type of sample collected was used to define the links between specific clusters. The MIRU-VNTR profile was analysed together with its corresponding spoligotype from this study and combined with results from previous studies in the province [18].

## Data Availability

The datasets used and analysed during the current study are available from the corresponding author on reasonable request.

## Abbreviations

bTB: Bovine tuberculosis
DRs: Direct repeats
HiP: Hluhluwe iMfolozi Park
KZN: KwaZulu-Natal
LJ: Löwenstein-Jensen
MIRU-VNTR: Mycobacteria interspersed repetitive unit-variable number of tandem repeats
MLVA: Multi locus VNTR analysis
PCR: Polymerase Chain Reaction
RD: Region of Differences
UPGMA: Unweighted pair-group method using average linkages algorithm
ZN: Ziehl Neelsen
